# Application Value of Predictive Model Based on Maternal Coagulation Function and Glycolipid Metabolism Indicators in Early Diagnosis of Gestational Diabetes Mellitus

**DOI:** 10.3389/fpubh.2022.850191

**Published:** 2022-03-21

**Authors:** Ying Zheng, Weiwei Hou, Jing Xiao, Hongling Huang, Wenqiang Quan, Yu Chen

**Affiliations:** ^1^Department of Surgery, Shanghai Jiao Tong University Affiliated Sixth People's Hospital, Shanghai, China; ^2^Department of Laboratory Medicine, Shanghai Tongji Hospital, School of Medicine, Tongji University, Shanghai, China; ^3^Department of Obstetrics and Gynecology, Shanghai Jiao Tong University Affiliated Sixth People's Hospital, Shanghai, China

**Keywords:** gestation, diabetes mellitus, fasting plasma glucose, coagulation function, lipid metabolism, prediction

## Abstract

**Objective:**

To investigate whether first-trimester fasting plasma glucose (FPG), blood coagulation function and lipid metabolism could predict gestational diabetes mellitus (GDM) risk.

**Methods:**

From October 2020 to May 2021, a total of 584 pregnant women who took prenatal care in Shanghai Jiaotong University Affiliated Sixth People's Hospital were chosen as the observation subjects. The clinical information and serum samples of all pregnant women were collected at 10–13 weeks of gestation and the blood coagulation function, fasting blood glucose and lipid profiles of the pregnant women were detected. A 75 g oral glucose tolerance test was performed up to 24–28 weeks of gestation. One hundred forty-two pregnant women with GDM and 442 pregnant women without GDM were detected. Data were expressed by x ± s or median (interquartile range) and were analyzed using student's *t*-test, Wilcoxon rank sum test and Logistic regression analysis. The area under the curve (AUC) was calculated by receiver operating characteristic curve (ROC) to analyze the predictive values.

**Results:**

Compared with non-GDM group, age, pre-pregnancy BMI, FPG, FIB, D-Dimer, FDP, FPG, TC, TG, LDL-C, sdLDL-C, APOB and APOE in GDM group were significantly higher than those in non-GDM group, while PT, INR, APTT and TT were significantly lower than those in non-GDM group. Univariate logistic regression analysis was used to explore the risk factors of GDM. Gestational age, pre-pregnancy BMI, FPG, PT, INR, APTT, FIB, TT, D-Dimer, TC, TG, LDL-C, sdLDL-C, APOB and APOE were all independent predictors of GDM. Multivariatelogistic regression showed that pre-pregnancy BMI, FPG, APTT, TT, TG, LDL-C, sdLDL-C and APOB were risk factors for GDM. The AUC of the established GDM risk prediction model was 0.892 (0.858–0.927), and the sensitivity and specificity were 80.71 and 86.85%, respectively; which were greater than that of pre-pregnancy BMI, FPG, APTT, TT,TG, LDL-C, sdLDL-C, APOB alone, and the difffference was statistically signifificant (*P* < 0.05).

**Conclusions:**

FPG, APTT, TT, TG, LDL-C, sdLDL-C, APOB and pre-pregnancy BMI in early pregnancy has important clinical value for the prediction of GDM, We combined these laboratory indicators and established a GDM risk prediction model, which is conducive to the early identification, intervention and treatment of GDM, so as to reduce the morbidity of maternal and infant complications.

## Introduction

Gestational diabetes mellitus (GDM) is a kind of impaired glucose metabolism that arises or is diagnosed during pregnancy, and it is one of the most prevalent pregnancy problems. The prevalence of GDM has risen steadily in recent years (1, 2). GDM has a number of negative consequences for both moms and their children. With the continuous progress in knowledge of GDM, most industrialized nations now test for GDM at 24–28 weeks of gestation (3). Early detection and treatment of GDM has been demonstrated in studies to enhance pregnancy outcomes (4, 5). However, the current unequivocal diagnosis of GDM is generally in the second trimester, thus the potential for early intervention and treatment may be missed. Now there is strong evidence that early diagnosis of GDM will allow for timely treatment, such as dietary counseling or lifestyle interventions, which has been shown to be effective for the improvement of perinatal outcomes (6). As a result, identifying risk variables and developing a simple and effective GDM risk prediction model, particularly in early pregnancy, has significant therapeutic application value.

Pregnant women's clotting function and lipid metabolism alter significantly as their pregnancy continues. The production of coagulation factors VII, VIII, IX, X, XII, and fibrinogen increase dramatically, peaking during a full-term pregnancy. The body's blood coagulation capability is strengthened, and it is in a particular physiological hypercoagulable condition, which might be lower the risk of postpartum hemorrhage (7). To maintain normal pregnancy needs and fetal growth and development, pregnant women's fat synthesis and blood lipid levels rise in the early stages of pregnancy owing to excessive phagocytosis and increased insulin sensitivity (8). But whether this increase is natural or pathological, few studies have been conducted to determine if it may be utilized as a possible clinical signal to predict the risk of later GDM.

Previous research demonstrated that a comparative proteomic study of plasma proteins from pregnant women with GDM and normoglycemia revealed that the differences were mostly connected to the coagulation and complement pathways (9). Some researchers have discovered that hyperlipidemia increases coagulation activity and shortens prothrombin time in patients with high total cholesterol or triglycerides (10), and that poor blood glucose control negatively affects lipid metabolism and coagulation function in patients with diabetes-complicated pregnancy (11). As a result of the intertwined relationship between pregnancy, diabetes, the blood coagulation cascade, and lipid metabolism, it is worth further discussion whether it can be combined with commonly used clinical laboratory indicators such as coagulation function, blood sugar, and blood lipids to predict GDM in the early stage. This study intends to establish a prospective follow-up cohort to collect general data such as pregnant women's ages, pre-pregnancy BMI, as well as early pregnancy coagulation function and glycolipid metabolism indicators, and then use logistic regression to establish a GDM prediction model and evaluate its effectiveness. The goal of this project is to make it feasible to recognize, diagnose, and intervene in GDM in the clinic as early as possible.

## Materials and Methods

### Patients

As the observation objects for prospective cohort research, we chose 584 pregnant women who had their first birth check-up card at Shanghai Jiaotong University Affiliated Sixth People's Hospital between October 2020 and May 2021. When the card was formed at 10–13 weeks of pregnancy, clinical information and peripheral blood samples were obtained from all pregnant women. The 75 g oral glucose tolerance test was performed during 24–28 weeks of pregnancy. There were 142 instances of GDM pregnant women and 442 cases of non-GDM pregnant women found. GDM diagnosis criteria include: Adopt the IADPSG-recommended GDM diagnostic approach, which is to test for GDM between 24 and 28 weeks of gestation. Pregnant women are given an oral glucose tolerance test of 75 g. If you have fasting blood glucose ≥5.1 mmol/L or oral glucose 1 h later, blood glucose ≥10.0 mmol/L or oral glucose 2 h after fasting blood glucose ≥8.5 mmol/L, might be diagnosed with GDM (12). Excluding numerous pregnancies, diabetes during pregnancy, hypertension, thyroid illness, cardiovascular disease, liver and kidney disease, autoimmune disease, and any other medical history of conditions impacting glucose and lipid metabolism. The Ethics Committee of Shanghai Sixth People's Hospital accepted an informed consent form completed by all observation subjects (Approval No. 2016-003).

### Clinical Information and Laboratory Examination

Baseline clinical data from 584 enrolled individuals' medical records were obtained, including age, and Body Mass Index (BMI) before pregnancy. The enrolled patients fasted after 22 p.m. in the evening of the day before the blood draw, and peripheral venous whole blood was drawn at 8 a.m. in the morning of the following day, centrifuged at 3,000 rpm for 10 min, and serum or plasma was obtained. Automated coagulation function analyzers (Siemens, Germany) and automated biochemical analyzers (Beckman, USA) were used to investigate and statistically analyze these laboratory data. The biochemical parameters from coagulation function, fasting plasma glucose, blood lipid and lipoprotein profiles examinations were collected by Automated coagulation function analyzers (Siemens, Germany) and automated biochemical analyzers (Beckman, USA), as shown in Table 1.

**Table 1:** List of variables collected from coagulation function, fasting plasma glucose, blood lipid and lipoprotein profiles examinations.

**Measured variables (abbreviation, SI)**	**Range of reference values**
Prothrombin time (PT, s)	11–14
International standardized ratio (INR)	0.82–1.15
Activated partial thromboplastin time (APTT, s)	23.3–32.5
Fibrinogen (FIB, g/L)	2–4
Thrombin time (TT, s)	13–21
D-Dimer measurement (D-Dimer, mg/L)	0–0.8
Fibrin degradation product (FDP, mg/L)	0–5
Fasting blood glucose (FPG, mmol/L)	4.1–5.9
Total cholesterol (TC, mmol/L)	2.8–5.9
Triglycerides (TG, mmol/L)	0.45–1.81
High-density lipoprotein cholesterol (HDL-C, mmol/L)	>1.03
Low-density lipoprotein cholesterol (LDL-C, mmol/L)	<4.10
Small and dense low-density lipoprotein cholesterol (sdLDL-C, mg/L)	94–428
Apolipoprotein A1 (APOA-1, g/L)	1.04–2.02
Apolipoprotein B (APOB, g/L)	0.66–1.33
Apolipoprotein E (APOE, mg/L)	29–53
Lipoprotein (a) (LPa, mg/dL)	0–30

### Statistical Analysis

SPSS 25.0 was used to do statistical analysis on the data that matched the criteria, and the Kolmogorov–Smirnov test was utilized to perform normal test analyses on the measurement data. The standard deviation of normally distributed data is given as x ± standard deviation (SD). The *t*-test (data conforms to a normal distribution and variance homogeneity) or Wilcoxon rank-sum test (not conforms to normal distribution and homogeneity of variance) was performed to compare the two groups. The skewed distribution measurement data are displayed as the median (M) and interquartile range (IQR). Independent sampling was used to compare skewed distribution measurement data using the Kruskal–Wallis H test.

The GraphPad Prism 8.0 software was used to create the receiver operating characteristic (ROC) curves for each indicator and combined test to determine the sensitivity, specificity, optimal cutoff value, Youden index, negative predictive value (NPV), and positive predictive value (PPV) of each index in GDM and non-GDM patients. The area under the curve (AUC) was used to evaluate the test's accuracy. A Univariate logistic regression analysis was used to screen GDM risk variables, and multivariate logistic regression analysis was utilized to develop a GDM prediction model. The *Z*-test was used to compare the area under the ROC curve of each marker and binary logistic regression analysis was used to establish the joint predictors of each index.

A nomogram based on the logistic regression model was constructed with R software (version 4.1.2). To assess the ability of the nomogram model to discriminate GDM patients, the area of ROC and 95% CIs were calculated. To analyze the agreement between nomogram predictions and actual observations, the Hosmer-Lemeshow tests were performed and calibration curves were created. Ten-Fold Cross-validation, Leave-one-out cross-validation, and Bootstraps of 1,000 resamples (with replacement) were applied to internally validate the stability of the model. Models were evaluated using discrimination and calibration, and discrimination was assessed by calculating the area under the receiver operating characteristic (ROC) curve (AUC) result for the predicted probability. The calibration degree of the prediction model refers to the consistency between the predicted probability and the actual observed value, and the calibration degree is displayed by the Hosmer-Lemeshow test and the calibration curve results.

The optimal cut-off value for each index was selected according to ROC curve, and binary logistic regression analysis was used to assess the risk of each index in GDM and non-GDM. Factors with statistical significance in the univariate analysis (*P* < 0.01) were included in the multivariate logistic regression analysis, and binary logistic regression analysis was performed to calculate the single factor, multivariate-adjusted odds ratio, and 95% confidence interval (CI) values based on maximum likelihood estimation. The difference was considered statistically significant when *P*-value was < 0.05.

## Results

### Characteristics of the Enrolled Pregnant Women Between GDM Group and Non-GDM Group

**Figure 1:**
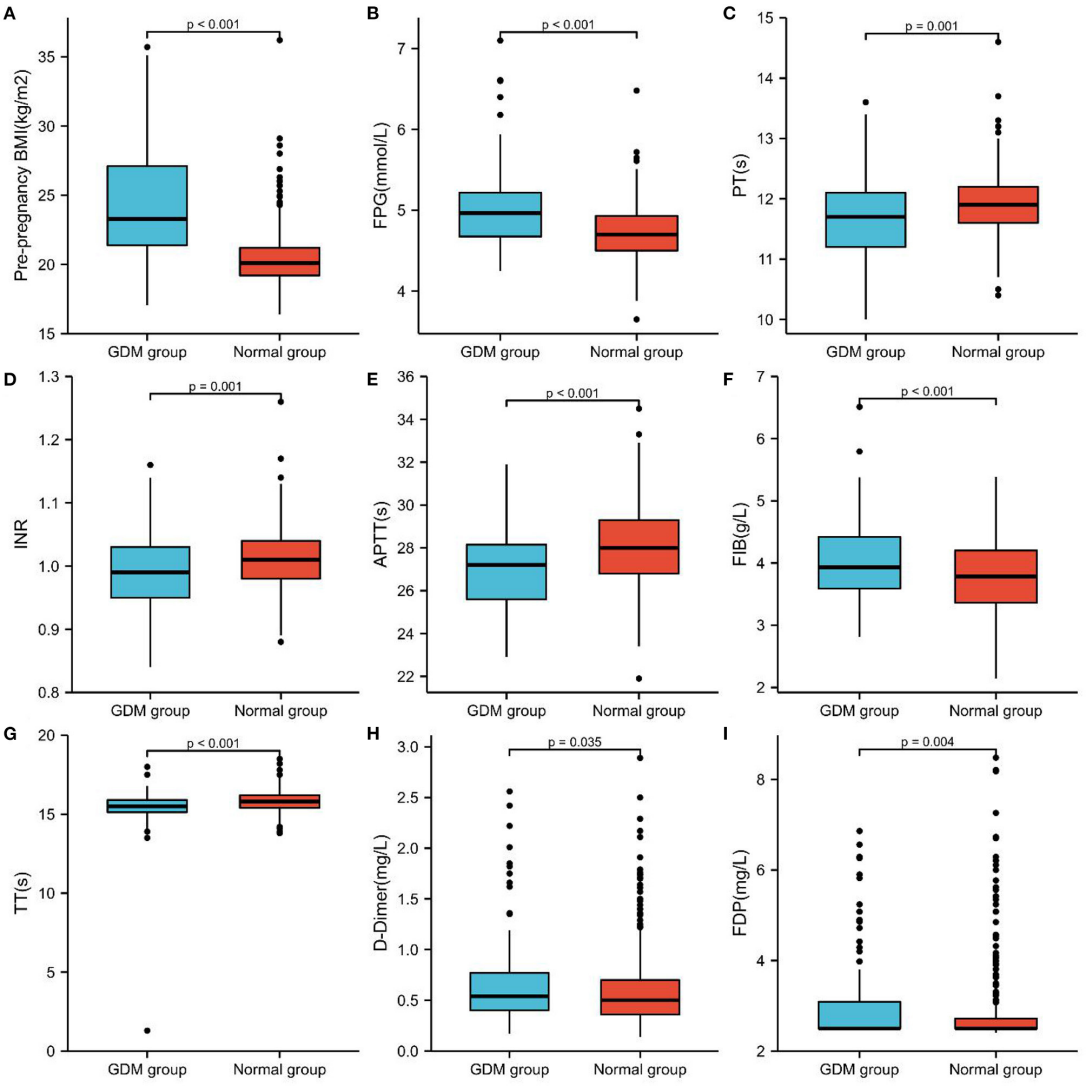
Comparison of Pre-pregnancy BMI, FPG and coagulation function of the two groups in pregnant women with GDM and non-GDM. **(A)** Pre-pregnancy BMI, **(B)** FPG, **(C)** PT, **(D)** INR, **(E)** APTT, **(F)** FIB, **(G)** TT, **(H)** D-D dimer, and **(I)** FDP.

**Figure 2:**
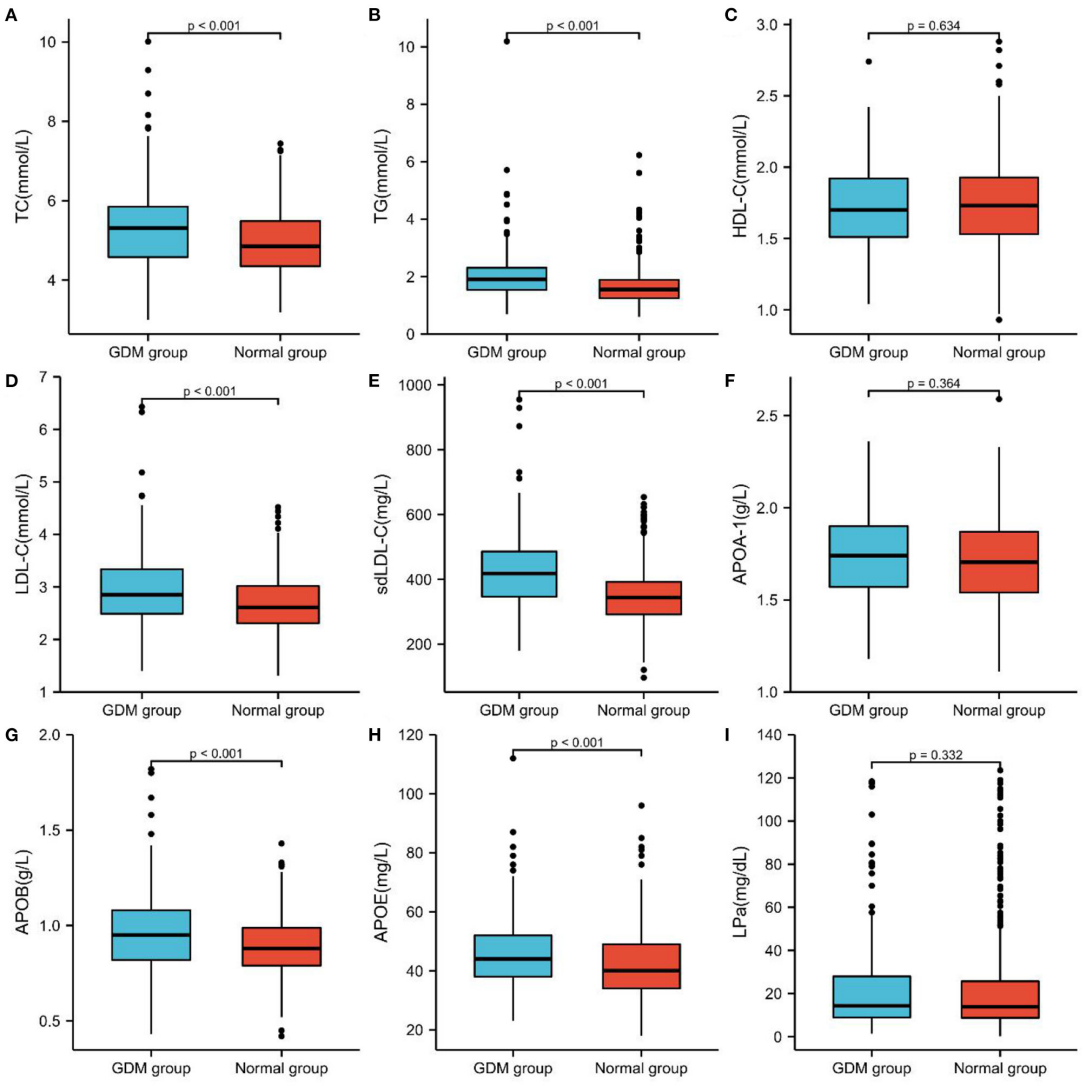
Comparison of blood lipid and lipoprotein profiles of the two groups in pregnant women with GDM and non-GDM. **(A)** TC, **(B)** TG, **(C)** HDL-C, **(D)** LDL-C, **(E)** sd LDL-C, **(F)** APOA-1, **(G)** APOB, **(H)** APOE, **(I)** LPa.

One hundred forty-two Pregnant Women Were Diagnosed With GDM in the second Trimester Among the 584 Pregnant Women Recruited in the Observation Group, and the Incidence of GDM Was 24.31%. The GDM Group's age, pre-Pregnancy BMI, FPG, FIB, D-D Dimer, FDP, FPG, TC, TG, LDL-C, sd LDL-C, APOB, and APOE Levels Were Considerably Greater Than Those of the non-GDM Group. While the PT, INR, APTT, and TT Indicators of the GDM Group Were Much Lower Than Those of the non-GDM Group, and the Difference Was Statistically Significant (*P* < 0.05), There Was no Significant Difference in the HDL-C, APOA-1, and LPa Between These two Groups (Table 2; Figures 1, 2).

**Table 2:** Comparison of basic clinical data of the two groups in pregnant women.

**Indicators**	**GDM group (*n* = 142)**	**Normal group (*n* = 442)**	***t*** **/Z value**	***P*** **-value**
Age (y)^*^	30.38 ± 4.33	29.08 ± 4.31	3.130	0.002
Pre-pregnancy BMI (kg/m^2^)	23.29 (21.38, 27.1)	20.1 (19.2, 21.2)	11.37	0.000
PT (s)	11.7 (11.2, 12.1)	11.9 (11.6, 12.2)	3.186	0.001
INR	0.99 (0.95, 1.03)	1.01 (0.98, 1.04)	3.269	0.001
APTT (s)^*^	26.92 ± 1.75	28.12 ± 1.86	−6.767	0.000
FIB (g/L)	3.933 (3.588, 4.421)	3.784 (3.362, 4.202)	3.583	0.000
TT (s)	15.5 (15.125, 15.9)	15.8 (15.4, 16.2)	5.153	0.000
D-Dimer (mg/L)	0.54 (0.40, 0.77)	0.50 (0.36, 0.70)	2.109	0.035
FDP (mg/L)	2.5 (2.5, 3.09)	2.5 (2.5, 2.72)	2.850	0.004
FPG (mmol/L)	4.965 (4.675, 5.218)	4.7 (4.5, 4.93)	7.237	0.000
TC (mmol/L)	5.31 (4.58, 5.85)	4.85 (4.35, 5.487)	3.655	0.000
TG (mmol/L)	1.91 (1.54, 2.31)	1.55 (1.25, 1.89)	6.403	0.000
HDL- (mmol/L)	1.7 (1.51, 1.92)	1.73 (1.53, 1.928)	0.477	0.634
LDL-C (mmol/L)	2.85 (2.49, 3.34)	2.61 (2.31, 3.018)	4.831	0.000
sdLDL-C (mg/L)	418.3 (346.75, 485.975)	344.15 (291.85, 392.1)	7.492	0.000
APOA-1 (g/L)^*^	1.73 ± 0.25	1.71 ± 0.23	0.909	0.364
APOB (g/L)	0.95 (0.82, 1.08)	0.88 (0.79, 0.987)	3.693	0.000
APOE (mg/L)	44 (38, 52)	40 (34, 49)	4.445	0.000
LPa (mg/dL)	14.3 (8.9, 29.6)	13.9 (8.67, 25.72)	0.970	0.332

*BMI, body mass index; PT, prothrombin time; INR, international standardized ratio; APTT, activated partial thromboplastin time; FIB, fibrinogen; TT, thrombin time; D-Dimer, D-dimer determination; FDP, fibrin degradation products; FPG, fasting blood glucose; TC, total cholesterol; TG, triglycerides; HDL-C, high density lipoprotein; LDL-C, low density lipoprotein; sdLDL-C, small and low density Density lipoprotein cholesterol; APOA-1, Apolipoprotein A1; APOB, Apolipoprotein B; APOE, Apolipoprotein E; LPa, Lipoprotein (a);*

* *The results of normality test showed that the observed variables were close to normal distribution in each group*.

### Univariate Logistic Regression Analysis of Risk Factors for GDM

An investigation of the risk variables for GDM was carried out using univariate logistic regression analysis. As shown in Table 3, age, pre-pregnancy BMI, FPG, PT, INR, APTT, FIB, TT, D-Dimer, TC, TG, LDL-C, sdLDL-C, APOB, and APOE were all predictors of gestational diabetes in the study population (*P* < 0.05).

**Table 3:** Univariate logistic regression analysis results of predictors of GDM.

**Indicators**	***B*** **value**	***SEM***	***Wald***	***P-*** **value**	***OR***	**95% *CI* lower**	**95% *CI* higher**
Age	0.070	0.023	9.499	0.002	1.073	1.026	1.122
Pre-pregnancy BMI	0.459	0.047	95.923	0.000	1.583	1.444	1.736
FPG	2.176	0.301	52.342	0.000	8.814	4.888	15.894
PT	−0.569	0.183	9.614	0.002	0.566	0.395	0.811
INR	−6.424	2.035	9.970	0.002	0.002	0.000	0.087
APTT	−0.367	0.059	39.416	0.000	0.693	0.618	0.777
FIB	0.666	0.167	15.851	0.000	1.946	1.402	2.702
TT	−0.768	0.163	22.284	0.000	0.464	0.337	0.638
D-Dimer	0.468	0.233	4.057	0.044	1.597	1.013	2.520
TC	0.477	0.107	19.838	0.000	1.611	1.306	1.987
TG	0.764	0.140	29.878	0.000	2.147	1.633	2.824
LDL-C	0.890	0.165	29.220	0.000	2.434	1.763	3.360
sdLDL-C	0.007	0.001	49.810	0.000	1.007	1.005	1.009
APOB	2.366	0.539	19.253	0.000	10.656	3.703	30.661
APOE	0.033	0.008	17.433	0.000	1.034	1.018	1.050

### Construction of a Multivariate Logistic Regression Model for GDM Early Detection

The pregnant women group logit (P) (GDM group = 1, Non GDM group = 0) was regarded the dependent variable, with variables having *P* < 0.01 in the univariate logistic regression analysis, indicating that the pre-pregnancy BMI (X1), FPG (X2), APTT (X3), TT (X4), TG (X5), LDL-C (X6), sdLDL-C (X7), APOB (X8) were considered self variables. The predictive parameters that integrate these indicators were calculated using multivariate logistic regression analysis (Table 4). The regression equation was logit (P) = −7.101+0.401X1+1.596X2-0.233X3-0.387X4+0.553X5+ 1.814X6+0.010X7-8.715X8, with the joint predictor being the analysis result of numerous joint test indicators.

**Table 4:** Multivariate logistic regression analysis results of predictors of GDM.

**Indicators**	***B*** **value**	***SEM***	***Wald***	***P*** **-value**	***OR***	**95% *CI* lower**	**95% *CI* higher**
Pre-pregnancy BMI	0.401	0.054	54.778	0.000	1.494	1.343	1.662
FPG	1.596	0.384	17.289	0.000	4.934	2.325	10.471
APTT	−0.233	0.077	9.245	0.002	0.792	0.682	0.921
TT	−0.387	0.171	5.140	0.023	0.679	0.486	0.949
TG	0.553	0.190	8.439	0.004	1.739	1.197	2.526
LDL-C	1.814	0.699	6.731	0.009	6.136	1.558	24.161
sdLDL-C	0.010	0.003	12.967	0.000	1.010	1.005	1.016
APOB	−8.715	2.457	12.578	0.000	0.000	0.000	0.020

### The Diagnostic Value of Laboratory Indicators and the Established GDM Risk Prediction Model for GDM

**Figure 3:**
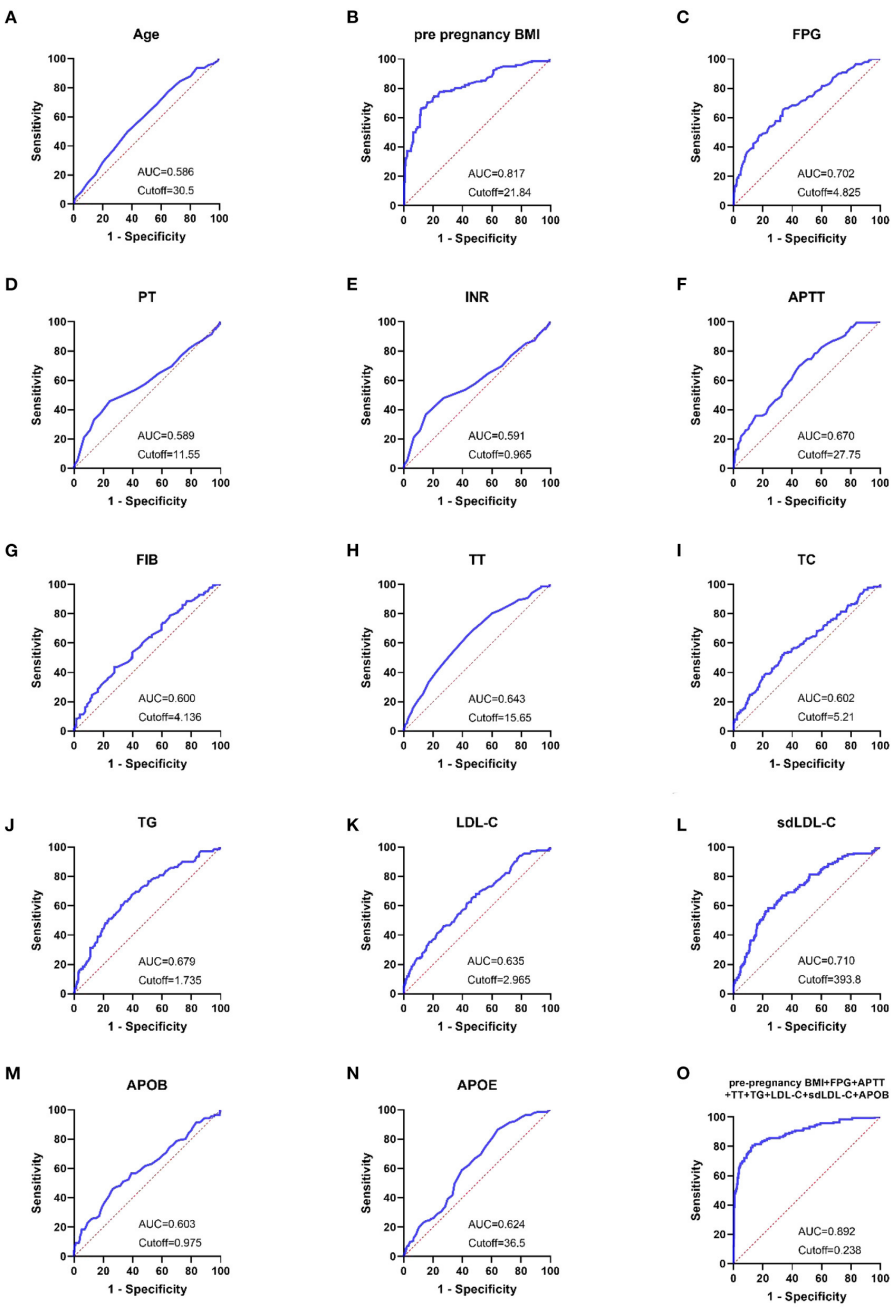
**(A–O)** ROC curves showed the diagnostic value of laboratory-related indicators for pregnant women with GDM.

The ROC curves for each indication and combination test were created using the GraphPad Prism program, as illustrated in Figures 3A–O. Pre-pregnancy BMI, FPG, and sdLDL-C were the single markers with the highest diagnostic value. When the threshold was 21.84 kg/m^2^, the AUC of pre-pregnancy BMI was 0.817, and the sensitivity, specificity, NPV, and PPV were 70.42, 83.94, 58.48, and 89.83 %, respectively. When the threshold was 4.825 mmol/L, the AUC of FPG was 0.702, and the sensitivity, specificity, NPV, and PPV were 65.49, 66.29, 38.42, and 85.67%, respectively. When the threshold was 393.8 mg/L, the AUC of sdLDL-C was 0.71, and the sensitivity, specificity, NPV, and PPV were 58.57, 76.24, 44.19, and 85.14%, respectively. When the threshold was 0.238, the AUC of the combined detection was 0.892. The combined detection's sensitivity, specificity, NPV, and PPV were 80.71, 86.85, 66.43, and 93.34%, respectively (In Table 5).

**Table 5:** Diagnostic performance of laboratory-related indicators in pregnant women with GDM.

**Indicators**	**Youden index**	**Cutoff**	**AUC**	**AUC 95% CI**	**Sensitivity**	**Specificity**	**PPV (%)**	**NPV (%)**
Age	0.1277	30.5	0.586	0.5332–0.6394	49.65	63.12	30.19	79.61
Pre-pregnancy BMI	0.5436	21.84	0.817	0.7734–0.8604	70.42	83.94	58.48	89.83
FPG	0.3178	4.825	0.702	0.6510–0.7524	65.49	66.29	38.42	85.67
PT	0.2151	11.55	0.589	0.5300–0.6473	45.77	75.74	37.73	81.30
INR	0.2165	0.965	0.591	0.5322–0.6496	36.62	85.03	44.00	80.68
APTT	0.2482	27.75	0.670	0.6195–0.7187	69.72	55.1	33.28	85.00
FIB	0.16	4.136	0.600	0.5464–0.6533	43.66	72.34	33.64	79.99
TT	0.2171	15.65	0.643	0.5914–0.6954	64.79	56.92	32.57	83.43
TC	0.1857	5.21	0.602	0.5469–0.6574	53.19	65.38	33.04	81.30
TG	0.2828	1.735	0.679	0.6280–0.7299	63.12	65.16	36.78	84.62
LDL-C	0.1895	2.965	0.635	0.5823–0.6877	46.1	72.85	35.29	80.80
sdLDL-C	0.3481	393.8	0.710	0.6605–0.7594	58.57	76.24	44.19	85.14
APOB	0.1963	0.975	0.603	0.5472–0.6592	46.1	73.53	35.87	80.94
APOE	0.2249	36.5	0.624	0.5747–0.6736	86.52	35.97	30.26	89.26
Combined test	0.6756	0.238	0.892	0.8581–0.9268	80.71	86.85	66.43	93.34

According to the findings in Table 4, the AUC values of combined detection were higher, and the diagnostic performance was greater. The AUC values of pre-pregnancy BMI, FPG, APTT, TT,TG, LDL-C, sdLDL-C, APOB, and pre-pregnancy BMI +FPG + APTT + TT + TG + LDL-C + sdLDL-C + APOB were compared using MedCalc software. The combined detection AUC was larger than that of pre-pregnancy BMI, FPG, APTT, TT, TG, LDL-C, sdLDL-C, and APOB alone, and the difference was statistically significant (*P* < 0.05; Table 6).

**Table 6:** Comparison of AUC areas for pre pregnancy BMI, FPG, APTT, TT, TG, LDL-C, sdLDL-C, APOB and combined test in GDM and non-GDM group.

**Detection indicators**	***Z*** **value**	***P*** **-value**
Combined test and pre-pregnancy BMI	3.950	0.0001
Combined test and FPG	7.940	<0.0001
Combined test and APTT	8.749	<0.0001
Combined test and TT	8.876	<0.0001
Combined test and TG	7.440	<0.0001
Combined test and LDL-C	8.761	<0.0001
Combined test and sdLDL-C	6.762	<0.0001
Combined test and APOB	8.972	<0.0001

### Establishment a Nomogram for Predicting GDM Based on Multivariable Logistic Regression Model

**Figure 4:**
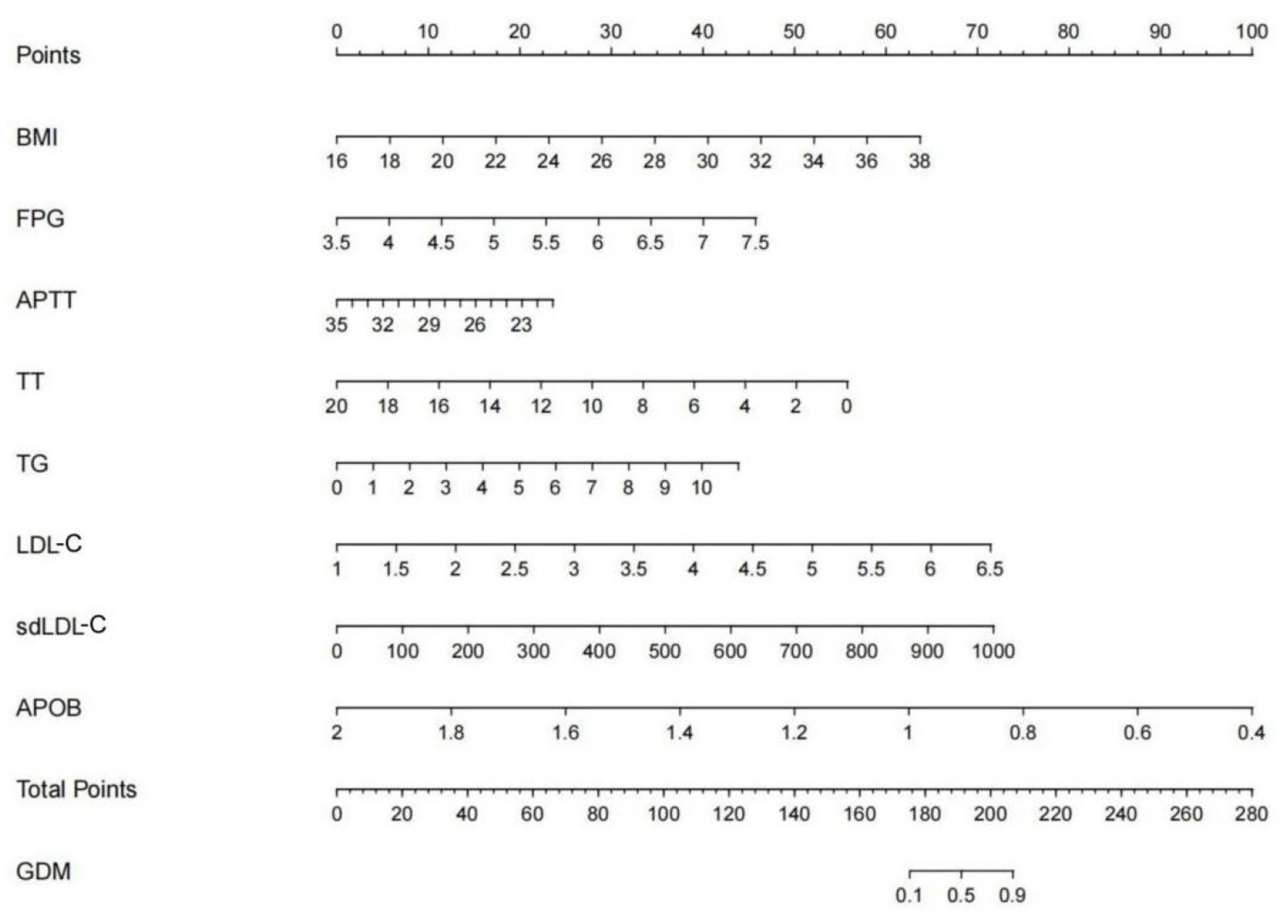
The nomogram of predictive model for GDM. Patient prognostic values were located on the axis of each variable. A vertical line was then drawn from that value to the top points scale to determine the number of points for that particular variable. The sum of these numbers was located on the total score axis, and a line was drawn at a 90° angle downward to the GDM risk axis to determine the risk of GDM.

Based on the multivariable model, a nomogram was generated. According to the data of pregnant women, read the corresponding points of pregnant women in this variable on the horizontal axis of each variable in the figure, and the value of the corresponding point of each variable perpendicular to the point on the axis marked with “score” is the score of this variable, The sum of the scores for each variable is the total score. Find the corresponding point of the total score on the “Total Score” axis, and the value of the point perpendicular to the “GDM” axis is the predicted probability of GDM. For example, using the developed nomogram, a pregnancy woman with pre-pregnant BMI of 26.4 kg/m2 (31 points), FPG of 4.87 mmol/L (16 points), APTT of 25.6S (15 points), TT of 15.2s (13 points),TG of 2.17 mmol/L (8 points), LDL of 2.45 mmol/L (17 points), sdLDL of 345 mg/L (25 points), APOB of 0.81 g/L (75 points), receives a total score of 200 points. The nomogram indicates that this pregnant woman may have a predictive probability of GDM of 0.81(Figure 4).

### Discrimination, Calibration Evaluation and Internal Validation of GDM Risk Prediction Model

**Figure 5:**
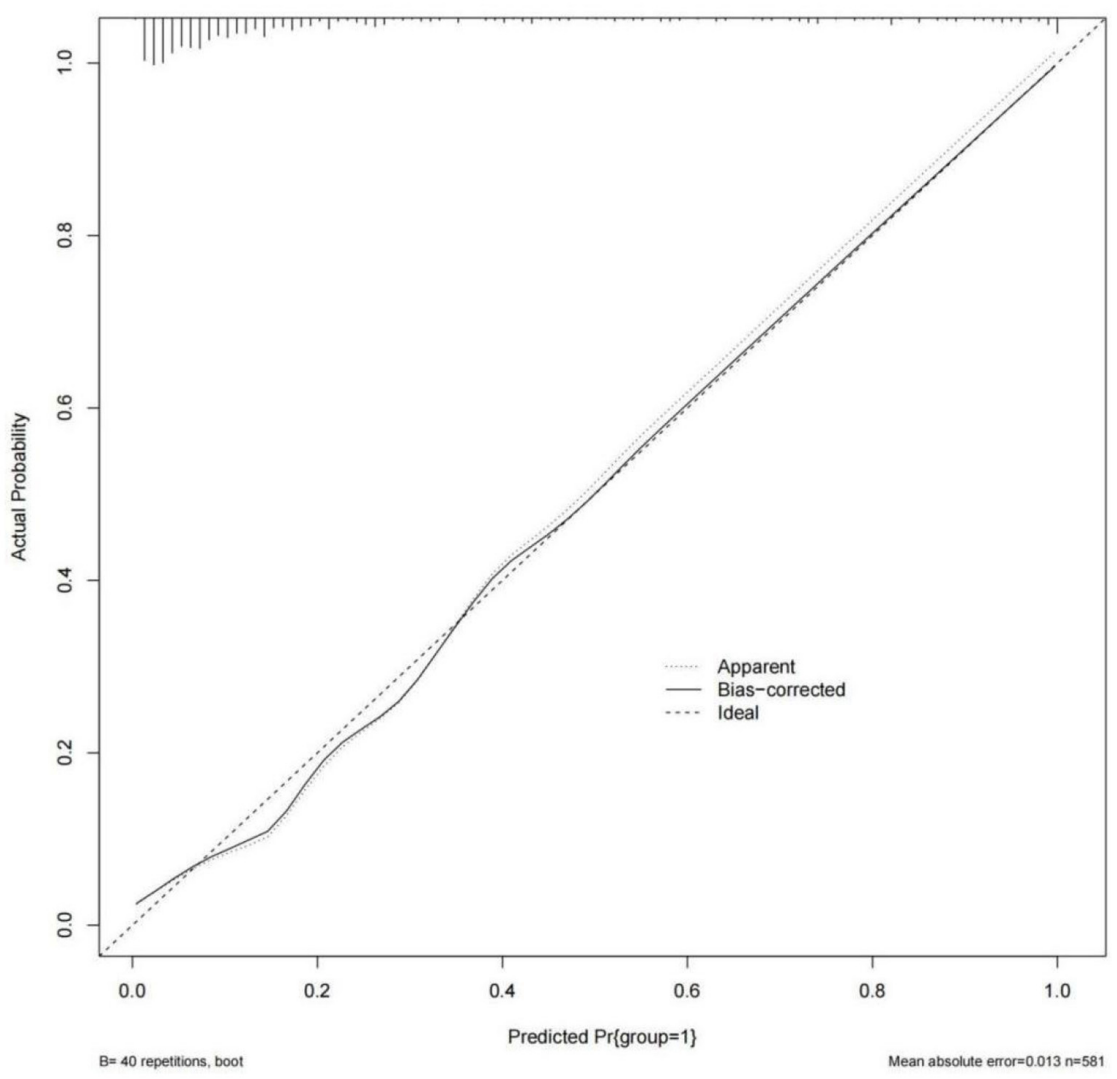
Calibration curve of GDM observation probability and prediction probability.

Model was evaluated using discrimination and calibration, and discrimination was assessed by calculating the area under the receiver operating characteristic (ROC) curve (AUC) result for the predicted probability. The AUC value of the model was 0.892 (95% CI: 0.858–0.927), indicating that the prediction model had a good degree of discrimination (Figure 3O). The Hosmer-Lemeshow test results showed that there was no statistical significance difference between the predicted risk value of the model and the actual observed value (χ^2^ = 6.022, *P* = 0.645). The Calibration curve showed that the predicted probability of the model was in good agreement with the actual probability, as shown in Figure 5. Cross-validation, Jackknife validation (cross-validation) and bootstrap sampling method (repetitive sampling 1,000 times) were used to conduct internal validation on the model data, and the C-statistics were 0.884, 0.882, and 0.892, respectively. The results are stable in the validation method, indicating that the model has good predictive performance in the modeling population.

### Pre-pregnancy BMI, FPG, APTT, TT,TG, LDL-C, sdLDL-C, and APOB Risk Assessment in Predicting GDM

**Figure 6:**
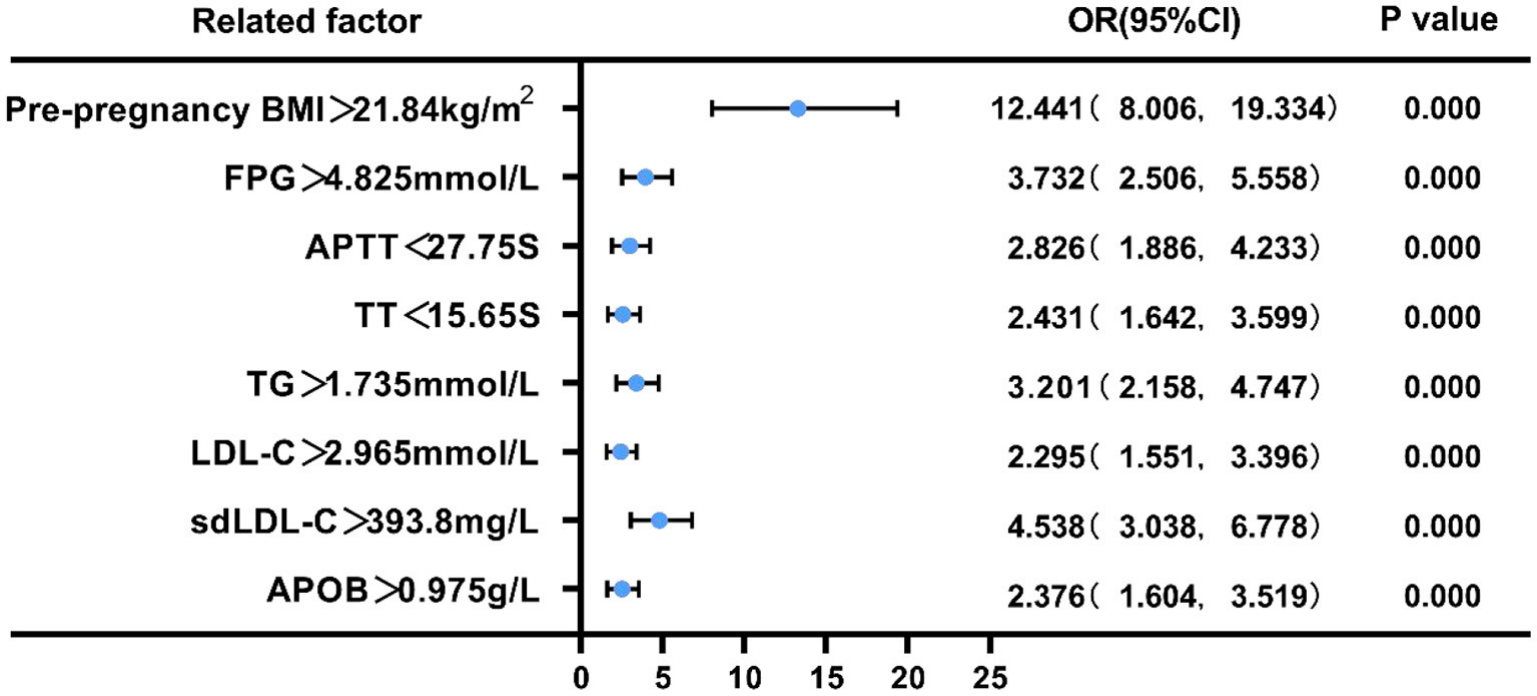
Forest plot of the univariate logistic regression analysis of pre-pregnancy BMI, FPG, APTT, TT,TG, LDL-C, sdLDL-C, APOB in pregnant women with GDM.

**Figure 7:**
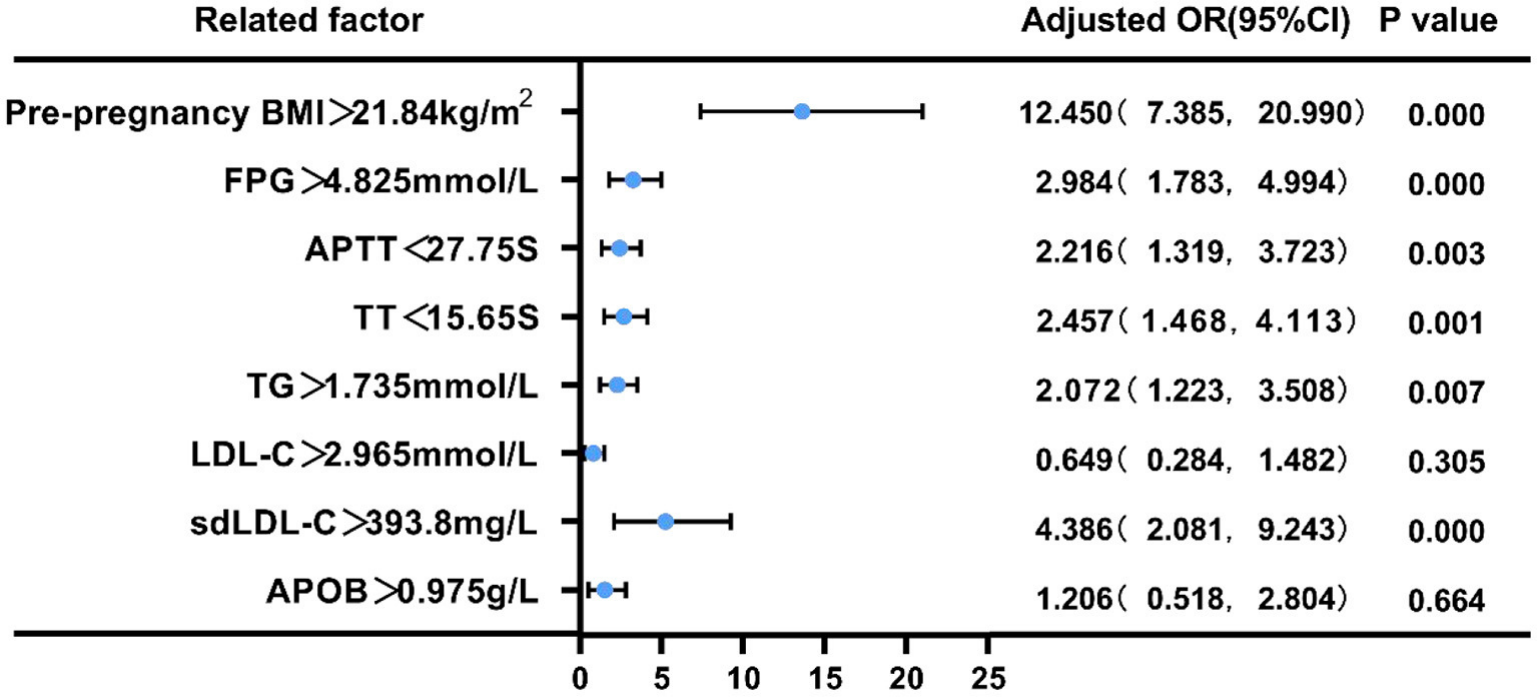
Forest plot of the multivariate logistic regression analysis of pre-pregnancy BMI, FPG, APTT, TT,TG, LDL-C, sdLDL-C, APOB in pregnant women with GDM.

We employed binary logistic regression analysis to assess the risk predictive value of pre-pregnancy BMI, FPG, APTT, TT, TG, LDL-C, sdLDL-C, and APOB levels in pregnant women with GDM. The cut-off value for each index was selected according to ROC curve. First, patients were split into two groups based on their pre-pregnancy BMI (21.84 kg/m^2^), FPG (4.825 mmol/L), APTT (27.75s), TT (15.65s), TG (1.735 mmol/L), LDL-C (2.965 mmol/L), sdLDL-C (393.8 mg/L), and APOB(0.975 g/L). Compared with low pre-pregnancy BMI, the risk of GDM in pregnant women with high pre- pregnancy BMI was 12.441 (95% CI = 8.006–19.334, *p* < 0.01), and the adjusted OR was 12.45 (95% CI = 7.385–20.99); Similarly, compared with low FPG, the risk of GDM in pregnant women with high FPG was 3.732 (95% CI = 2.506–5.558, *p* < 0.01), and the adjusted OR was 2.984 (95% CI = 1.783–4.994); Compared with prolonged APTT, the risk of GDM in pregnant women with shortened APTT was 2.826 (95% CI = 1.886–4.233, *p* < 0.01), and the adjusted OR was 2.216 (95% CI = 1.319–3.723); Compared with prolonged TT, the risk of GDM in pregnant women with shortened TT was 2.431 (95% CI = 1.4642–3.599, *p* < 0.01), and the adjusted OR was 2.457 (95% CI = 1.468–4.113); Compared with low TG, the risk of GDM in pregnant women with high TG was 3.201 (95% CI = 12.158–4.747, *p* < 0.01), and the adjusted OR was 2.072 (95% CI = 1.223–3.508); Compared with low LDL-C, the risk of GDM in pregnant women with high LDL-C was 2.295 (95% CI = 1.551–3.396, *p* < 0.01), and the adjusted OR was 4.386 (95% CI = 2.081–9.243); Compared with low sdLDL-C, the risk of GDM in pregnant women with high sdLDL-C was 4.538 (95% CI = 3.038–6.778, *p* < 0.01), and the adjusted OR was 0.649 (95% CI = 0.284–1.482); Compared with low APOB, the risk of GDM in pregnant women with high APOB was 2.376 (95% CI = 1.604–3.519, *p* < 0.01), and the adjusted OR was 1.206 (95% CI = 0.518–2.804) (*P* < 0.05, Figures 6, 7).

## Discussion

As a pregnancy complication, GDM is associated with glucose intolerance and insulin resistance (13). GDM is identified when an OGTT test is conducted at 24–28 weeks of pregnancy in women who do not have a history of GDM or diabetes mellitus before pregnancy. The blood glucose metabolism of most GDM patients will recover to normal after delivery, however, some GDM patients may acquire type 2 diabetes as a result of their condition (T2DM) (14). In a short time, gestational diabetes mellitus (GDM) might raise the risk of preeclampsia, polyhydramnios, preterm labor, and ketoacidosis in expecting mothers (15). Diabetes, cardiovascular disease, and metabolic syndrome are long-term consequences for the mother (16). As a result, neonates with GDM have a higher risk of problems, such as birth damage, respiratory distress syndrome, hyperbilirubinemia, and hypoglycemia. Fetal hyperinsulinemia, and macrosomia may result from inadequate blood glucose management in pregnant women with GDM (15). GDM has long-term negative impacts on children, including an increased incidence of Type 2 diabetes and obesity (17).

Recent years have seen an increase in the number of research attempting to develop risk prediction models for gestational diabetes mellitus (GDM). Predicting GDM based on clinical and biological signs is the subject of many investigations, and various mathematical models have been developed (18–20). However, the majority of GDM risk prediction models described in the literature relied only on the fundamental characteristics of pregnant women, such as age, nationality, and pre-pregnancy body mass index (Pre-pregnancy BMI). Most studies that were described were also retrospective, which limits the clinical value of the findings. Pregnant women's age, pre-pregnancy BMI, and various coagulation and blood glucose and blood lipid indicators in early pregnancy were integrated into our research to predict the likelihood of GDM and identify the associated risk factors. Increased pre-pregnancy body mass index (BMI), FPG, TG, LDL-C, sdLDL-C, APOB, and shorter APTT, TT was observed to be associated with an increased risk of gestational diabetes. GDM risk prediction model was built using the receiver operating characteristic curve (ROC) as a measure of model efficacy. As a consequence, this model has a high diagnostic value. It is possible to predict gestational diabetes (GDM) based on the combination of these clinical signs.

Endothelial damage caused by high blood glucose activates the internal coagulation system in pregnant women with GDM (21, 22), and some studies have also shown that the level of coagulation factor XII in pregnant women with GDM was significantly higher than healthy pregnant women (23). The reason might be that endothelial damage caused by high blood glucose activates the internal coagulation system in pregnant women with GDM. We also found that among pregnant women with GDM, the PT and APTT were shorter and the FPG was higher in the first trimester, suggesting that the variations in blood coagulation and blood glucose between the two groups did not begin in the second trimester. First-trimester pregnancy blood glucose and coagulation function monitoring may be useful in predicting and diagnosing GDM.

Pregnant women with elevated lipid levels have a higher chance of developing GDM. When it comes to TC, TG, and LDL-C, pregnant women with GDM had higher levels than pregnant women without the condition (24). But the findings of the meta-analysis suggested that in addition to a rise in TG, cholesterol variations across various groups were not consistent. Insulin resistance was caused by hypertriglyceridemia, not hypercholesterolemia (25). This research demonstrated that in the early stages of pregnancy, the GDM group had substantially higher levels of TC, TG, LDL-C, sdLDL-C, APOB, and APOE than the control group. HDL-C, APOA-1, and LPa levels were not significantly different between the two groups. A substantial difference in blood lipid distribution between GDM and non-GDM groups was found in this study. Early in pregnancy, GDM pregnant women suffer from more severe dyslipidemia and insulin resistance. We studied the correlation between early pregnancy blood lipid profile and GDM to discover whether dyslipidemia in early pregnancy had clinical prognostic implications. We found that when TG >1.735 mmol/L, LDL-C >2.965 mmol/L, sdLDL-C >393.8 mg/L, APOB >0.975 g/L, the incidence of GDM in pregnant women rose by 3.201, 2.295, 4.538, and 2.376 times, respectively. After adjustment OR the incidence of GDM in pregnant women rose by 2.072, 0.649, 4.386, 1.206 times, respectively. In the first trimester, TG, LDL-C, sdLDL-C and APOB may be excellent risk predictors of GDM.

By analyzing numerous coagulation, blood glucose, and blood lipid indicators in the early stages of pregnancy, it is possible to accurately predict the risk of GDM. This method and model of multiple indicators improves the sensitivity and specificity of prediction, helps to identify GDM early, and promotes early prevention, intervention, and treatment of GDM, thus improving pregnancy outcomes and reducing the risk of long-term metabolic diseases in pregnant women and their infants. However, there are several limitations to our research. First, it should be noted that the model needs external validation in an independent study. Second, this study is a single-center study with a relatively small sample size, which cannot yet represent the general significance in a large-scale clinical population. In addition, some clinical indicators found in the latest research were not included. therefore, larger sample sizes and joint survey studies from multiple centers may provide better clinical research value in the future.

## Data Availability Statement

The original contributions presented in the study are included in the article/supplementary material, further inquiries can be directed to the corresponding author/s.

## Ethics Statement

The studies involving human participants were reviewed and approved by the Ethics Committee of Shanghai Sixth People's Hospital (Approval No. 2016-003). The patients/participants provided their written informed consent to participate in this study. Written informed consent was obtained from the individual(s) for the publication of any potentially identifiable images or data included in this article.

## Author Contributions

YZ, WH, WQ, and YC contributed to study concept and design, acquisition of the data, analysis and interpretation of the data, and drafting of the manuscript. WQ and YC contributed to statistical analysis and contributed to funding acquisition. YZ and JX contributed to sample collections. WH and HH contributed to study supervision and critical revision of the manuscript. YC contributed to project administration. All authors have read and agreed to the published version of the manuscript.

## Funding

This work was supported by the Scientific Research Project of Shanghai Municipal Health and Family Planning Commission (201640250); Shanghai Rising Stars of Medical Talents Youth Development Program–Clinical Laboratory Practitioner Program (2021-JY); the Gan Quan Xin Xing talent training program of Shanghai Tongji Hospital (HRBC2005).

## Conflict of Interest

The authors declare that the research was conducted in the absence of any commercial or financial relationships that could be construed as a potential conflict of interest.

## Publisher's Note

All claims expressed in this article are solely those of the authors and do not necessarily represent those of their affiliated organizations, or those of the publisher, the editors and the reviewers. Any product that may be evaluated in this article, or claim that may be made by its manufacturer, is not guaranteed or endorsed by the publisher.

## References

[1] Juan J Yang H. Prevalence, prevention, and lifestyle intervention of gestational diabetes mellitus in China. Int J Environ Res Public Health. (2020) 17:9517. 10.3390/ijerph17249517 33353136 PMC7766930

[2] Lavery JA Friedman AM Keyes KM Wright JD Ananth CV. Gestational diabetes in the United States: temporal changes in prevalence rates between 1979 and 2010. BJOG. (2017) 124:804–13. 10.1111/1471-0528.14236 27510598 PMC5303559

[3] Duran A Sáenz S Torrejón MJ Bordiú E Del Valle L Galindo M. Introduction of IADPSG criteria for the screening and diagnosis of gestational diabetes mellitus results in improved pregnancy outcomes at a lower cost in a large cohort of pregnant women: the St. Carlos gestational diabetes study. Diabetes Care. (2014) 37:2442–50. 10.2337/dc14-0179 24947793

[4] Wang C Wei Y Zhang X Zhang Y Xu Q Sun Y. A randomized clinical trial of exercise during pregnancy to prevent gestational diabetes mellitus and improve pregnancy outcome in overweight and obese pregnant women. Am J Obstet Gynecol. (2017) 216:340–51. 10.1016/j.ajog.2017.01.037 28161306

[5] Guo XY Shu J Fu XH Chen XP Zhang L Ji MX. Improving the effectiveness of lifestyle interventions for gestational diabetes prevention: a meta-analysis and meta-regression. BJOG. (2019) 126:311–20. 10.1111/1471-0528.15467 30216635

[6] Buelo AK Kirk A Lindsay RS Jepson RG. Exploring the effectiveness of physical activity interventions in women with previous gestational diabetes: a systematic review of quantitative and qualitative studies. Prev Med Rep. (2019) 14:100877. 10.1016/j.pmedr.2019.100877 31110933 PMC6510702

[7] James AH Rhee E Thames B Philipp CS. Characterization of antithrombin levels in pregnancy. Thromb Res. (2014) 134:648–51. 10.1016/j.thromres.2014.07.025 25087890

[8] Herrera E Ortega-Senovilla H. Disturbances in lipid metabolism in diabetic pregnancy - are these the cause of the problem? Best Pract Res Clin Endocrinol Metab. (2010) 24:515–25. 10.1016/j.beem.2010.05.006 20832733

[9] Mavreli D Evangelinakis N Papantoniou N Kolialexi A. Quantitative comparative proteomics reveals candidate biomarkers for the early prediction of gestational diabetes mellitus: a preliminary study. In Vivo. (2020) 34:517–25. 10.21873/invivo.11803 32111749 PMC7157887

[10] Kim JA Kim JE Song SH. Influence of blood lipids on global coagulation test results. Ann Lab Med. (2015) 35:15–21. 10.3343/alm.2015.35.1.15 25553275 PMC4272949

[11] Teliga-Czajkowska J Sienko J Zareba-Szczudlik J Malinowska-Polubiec A Romejko-Wolniewicz E Czajkowski K. Influence of glycemic control on coagulation and lipid metabolism in pregnancies complicated by pregestational and gestational diabetes mellitus. Adv Exp Med Biol. (2019) 1176:81–8. 10.1007/5584_2019_382 31069723

[12] International International Association of Diabetes Pregnancy Study Groups Consensus Panel Metzger BE Gabbe SG Persson B Buchanan TA Catalano PA. International association of diabetes and pregnancy study groups recommendations on the diagnosis and classification of hyperglycemia in pregnancy. Diabetes Care. (2010) 33:676–82. 10.2337/dc10-0719 20190296 PMC2827530

[13] McIntyre HD Catalano P Zhang C Desoye G Mathiesen ER Damm P. Gestational diabetes mellitus. Nat Rev Dis Primers. (2019) 5:47. 10.1038/s41572-019-0098-8 31296866

[14] Vounzoulaki E Khunti K Abner SC Tan BK Davies MJ Gillies CL. Progression to type 2 diabetes in women with a known history of gestational diabetes: systematic review and meta-analysis. BMJ. (2020) 369:m1361. 10.1136/bmj.m1361 32404325 PMC7218708

[15] Billionnet C Mitanchez D Weill A Nizard J Alla F Hartemann A. Gestational diabetes and adverse perinatal outcomes from 716,152 births in France in 2012. Diabetologia. (2017) 60:636–44. 10.1007/s00125-017-4206-6 28197657 PMC6518373

[16] Xiang AH Li BH Black MH Sacks DA Buchanan TA Jacobsen SJ. Racial and ethnic disparities in diabetes risk after gestational diabetes mellitus. Diabetologia. (2011) 54:3016–21. 10.1007/s00125-011-2330-2 22016046

[17] Murray SR Reynolds RM. Short- and long-term outcomes of gestational diabetes and its treatment on fetal development. Prenat Diagn. (2020) 40:1085–91. 10.1002/pd.5768 32946125

[18] Nombo AP Mwanri AW Brouwer-Brolsma EM Ramaiya KL Feskens EJM. Gestational diabetes mellitus risk score: a practical tool to predict gestational diabetes mellitus risk in Tanzania. Diabetes Res Clin Pract. (2018) 145:130–7. 10.1016/j.diabres.2018.05.001 29852237

[19] Kramer CK Campbell S Retnakaran R. Gestational diabetes and the risk of cardiovascular disease in women: a systematic review and meta-analysis. Diabetologia. (2019) 62:905–14. 10.1007/s00125-019-4840-2 30843102

[20] Sesmilo G Prats P Garcia S Rodríguez I Rodríguez-Melcón A Berges I. First-trimester fasting glycemia as a predictor of gestational diabetes (GDM) and adverse pregnancy outcomes. Acta Diabetol. (2020) 57:697–703. 10.1007/s00592-019-01474-8 31984438

[21] Liu Y Sun X Tao J Song B Wu W Li Y. Gestational diabetes mellitus is associated with antenatal hypercoagulability and hyperfibrinolysis: a case control study of Chinese women. J Matern Fetal Neonatal Med. (2020) 14:1–4. 10.1080/14767058.2020.1818202 32928010

[22] Lippi G Franchini M Targher G Montagnana M Salvagno GL Guidi GC. Epidemiological association between fasting plasma glucose and shortened APTT. Clin Biochem. (2009) 42:118–20. 10.1016/j.clinbiochem.2008.10.012 19014926

[23] Ozbasli E Takmaz O Karabuk E Gungor M. Comparison of factor XII levels in gestational diabetes, fetal macrosomia, and healthy pregnancies. BMC Pregnancy Childbirth. (2020) 20:752. 10.1186/s12884-020-03455-0 33267793 PMC7709445

[24] Shen H Liu X Chen Y He B Cheng W. Associations of lipid levels during gestation with hypertensive disorders of pregnancy and gestational diabetes mellitus: a prospective longitudinal cohort study. BMJ Open. (2016) 6:e013509. 10.1136/bmjopen-2016-013509 28011814 PMC5223699

[25] Ryckman KK Spracklen CN Smith CJ Robinson JG Saftlas AF. Maternal lipid levels during pregnancy and gestational diabetes: a systematic review and meta-analysis. BJOG. (2015) 122:643–51. 10.1111/1471-0528.13261 25612005

